# Comprehensive analysis of multi‐omics single‐cell data using the single‐cell analyst

**DOI:** 10.1002/imt2.70038

**Published:** 2025-04-28

**Authors:** Lu Pan, Bufu Tang, Xuan Zhang, Paolo Parini, Roman Tremmel, Joseph Loscalzo, Volker M. Lauschke, Bradley A. Maron, Paola Paci, Ingemar Ernberg, Nguan Soon Tan, Ákos Végvári, Zehuan Liao, Sundararaman Rengarajan, Roman Zubarev, Yuxuan Fan, Xu Zheng, Xinyue Jian, Ren Sheng, Zhenning Wang, Xuexin Li

**Affiliations:** ^1^ Institute of Environmental Medicine Karolinska Institutet Solna Sweden; ^2^ Department of Radiation Oncology Zhongshan Hospital Affiliated to Fudan University Shanghai China; ^3^ Department of Colorectal Surgery, Yunnan Cancer Hospital The Third Affiliated Hospital of Kunming Medical University Kunming Yunnan China; ^4^ Cardio Metabolic Unit, Department of Medicine, and Department of Laboratory Medicine Karolinska Institutet Stockholm Sweden; ^5^ University of Tuebingen Tuebingen Germany; ^6^ Department of Medicine, Brigham and Women's Hospital Harvard Medical School Boston Massachusetts USA; ^7^ Department of Physiology and Pharmacology Karolinska Institutet Solna Sweden; ^8^ Department of Computer, Control and Management Engineering Sapienza University of Rome Rome Italy; ^9^ Department of Microbiology, Tumor and Cell Biology Karolinska Institutet Solna Sweden; ^10^ School of Biological Sciences Nanyang Technological University Singapore Singapore; ^11^ Lee Kong Chian School of Medicine Nanyang Technological University Singapore Singapore Singapore; ^12^ Department of Medical Biochemistry and Biophysics Karolinska Institutet Solna Sweden; ^13^ Department of Physical Therapy, Movement & Rehabilitation Sciences Northeastern University Boston Massachusetts USA; ^14^ China Medical University‐The Queen's University of Belfast Joint College China Medical University Shenyang Liaoning China; ^15^ Biomedical Innovation Center, The Fourth Affiliated Hospital China Medical University Shenyang Liaoning China; ^16^ College of Life and Health Sciences Northeastern University Shenyang Liaoning China; ^17^ School of Basic Medical Sciences Guangzhou Medical University Guangdong China; ^18^ Key Laboratory of Precision Diagnosis and Treatment of Gastrointestinal Tumors, Ministry of Education China Medical University Shenyang Liaoning China; ^19^ Institute of Health Sciences China Medical University Shenyang Liaoning China; ^20^ The First Affiliated Hospital of China Medical University Shenyang Liaoning China; ^21^ Department of General Surgery, The Fourth Affiliated Hospital China Medical University Shenyang Liaoning China

**Keywords:** multi‐omics, single‐cell sequencing, web server

## Abstract

The rapid advancement of multi‐omics single‐cell technologies has significantly enhanced our ability to investigate complex biological systems at unprecedented resolution. However, many existing analysis tools are complex, requiring substantial coding expertize, which can be a barrier for computationally less competent researchers. To address this challenge, we present single‐cell analyst, a user‐friendly, web‐based platform to facilitate comprehensive multi‐omics analysis. Single‐cell analyst supports a wide range of data types, including six single‐cell omics: single‐cell RNA sequencing (scRNA‐sequencing), single‐cell assay for transposase accessible chromatin sequencing (scATAC‐seq sequencing), single‐cell immune profiling (scImmune profiling), single‐cell copy number variation, cytometry by time‐of‐flight, and flow cytometry and spatial transcriptomics, and enables researchers to perform integrated analyses without requiring programming skills. The platform offers both online and offline modes, providing flexibility for various use cases. It automates critical analysis steps, such as quality control, data processing, and phenotype‐specific analyses, while also offering interactive, publication‐ready visualizations. With over 20 interactive tools for intermediate analysis, single cell analyst simplifies workflows and significantly reduces the learning curve typically associated with similar platforms. This robust tool accommodates datasets of varying sizes, completing analyses within minutes to hours depending on the data volume, and ensures efficient use of computational resources. By democratizing the complex process of multi‐omics analysis, single‐cell analyst serves as an accessible, all‐encompassing solution for researchers of diverse technical backgrounds. The platform is freely accessible at www.singlecellanalyst.org.

## Highlights

- Single‐cell analyst is a web‐based platform for comprehensive multi‐omics single‐cell data analysis, supporting six single‐cell omics types and spatial transcriptomics.
- It eliminates the need for coding, offering an intuitive, user‐friendly interface accessible to researchers at all levels.
- The platform automates key analysis steps, including quality control, data processing, and phenotypic analysis, delivering interactive, publication‐ready visualizations.
- Single‐cell analyst streamlines workflows, making complex multi‐omics analyses accessible without requiring extensive computational expertize.

## Graphical abstract

Single‐cell analyst is a user‐friendly, web‐based platform designed for comprehensive multi‐omics single‐cell data analysis. Supporting six single‐cell omics types and spatial transcriptomics, it eliminates the need for coding, making advanced data analysis accessible to researchers of all expertize levels. The platform automates key analytical steps, including quality control, data processing, and phenotypic analysis, while generating interactive, publication‐ready visualizations. By streamlining complex multi‐omics workflows, single‐cell analyst empowers users to efficiently explore and interpret single‐cell data without requiring extensive computational skills.

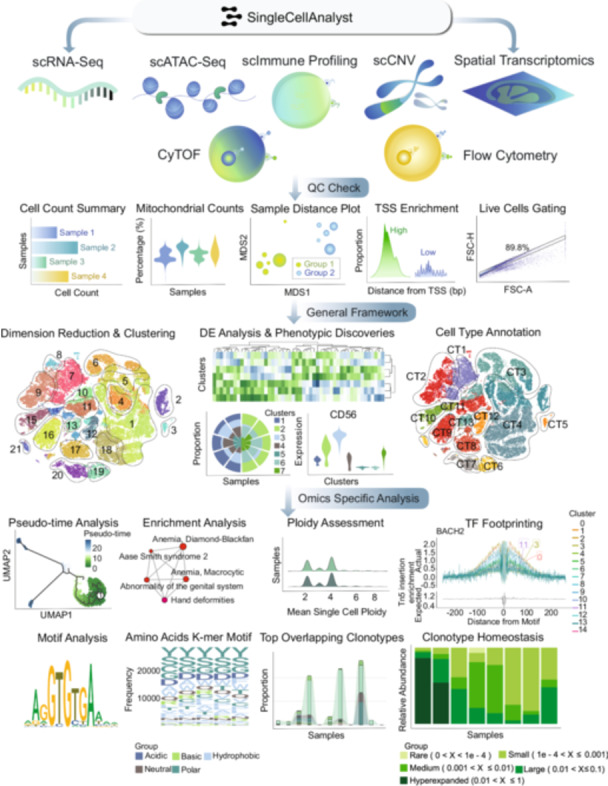

## INTRODUCTION

The advent of single‐cell omics technologies has amplified our capacity to examine biological phenomena at the single‐cell level across diverse molecular layers and has catalyzed an exponential increase in single‐cell multi‐omics studies in recent years (Figure S1 and Table S1). This trend coincides with the steady evolution of new analysis methods and tools for interpreting these novel data forms [1, 2, 3]. However, existing platforms are often limited in scope and usability, catering primarily to single modalities like single‐cell (sc) RNA‐sequencing (scRNA‐seq) [4] and requiring significant coding expertize [2] (Figure S1). In the area of web‐based tools [5, 6, 7], there is currently a gap in the availability of comprehensive, coding‐free solutions that facilitate an entire suite of analysis workflows across multiple and between omics, especially for the current popular 10× Genomics omics types. These tools tend to focus on a limited number of omics, covering at most three single‐cell omics types (Table S1), whereas some exhibit high complexity for new users [3, 8].

**Figure 1:**
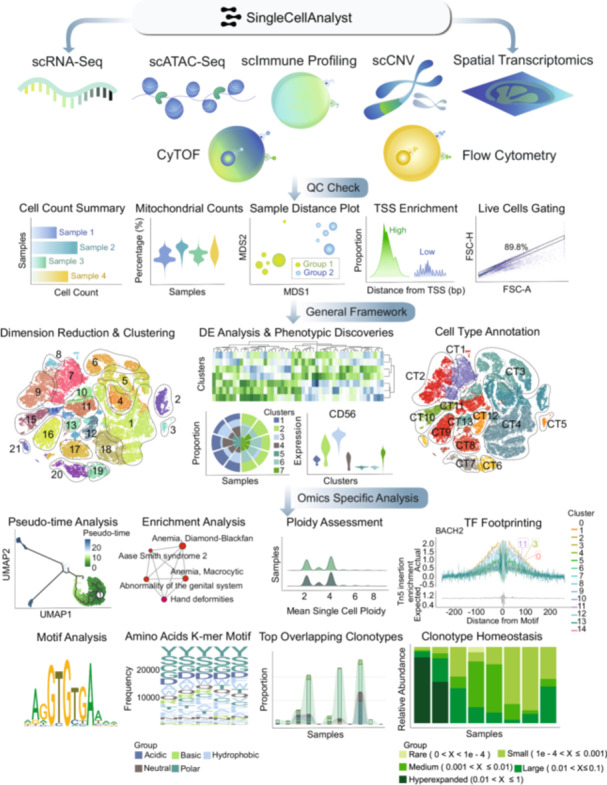
An overview of the analysis frameworks on the single cell analyst web server. Seven frameworks are available on the platform for users to carry out single‐cell multimodal analyses. Analyses for the omics types on the first row are mostly catered for 10× Genomics data formats. The analysis also supports data from other technologies as long as the stated data formats are provided. The first set of the framework included quality control (QC) steps, with omics‐specific QC checks such as automated gating for flow cytometry data. This will be followed by a general analysis framework, including post‐QC dimensionality reduction, clustering, DE analysis, and the prediction of cell types in each cluster. Next, omics‐specific analyses will be performed. Owing to the different natures of the data types from each omics data set, additional analyses will be carried out to meet omics‐specific analysis requirements. For example, pseudotime analyses will be carried out during an analysis of single‐cell RNA sequencing (scRNA‐seq) data. Clonal homeostasis will be carried out for single‐cell immune (scImmune Profiling) data to assess the spatial occupancies of clones across samples. BACH2, BTB and CNC Homology 2; CD56, cluster of differentiation 56; CT, cell type; CyTOF, cytometry by time‐of‐flight; DE analysis, differential expression analysis; FSC‐A, forward scatter area; MDS, multidimensional scaling; QC check, quality control check; scRNA‐seq, single‐cell RNA sequencing; scATAC‐seq, single‐cell assay for transposase accessible chromatin sequencing; scImmune profiling, single cell immune profiling; scCNV, single‐cell copy number variation; TSS, transcription start site; TF, transcription factor; TF footprinting, transcription factor footprinting; UMAP, uniform manifold approximation and projection.

**Figure 2:**
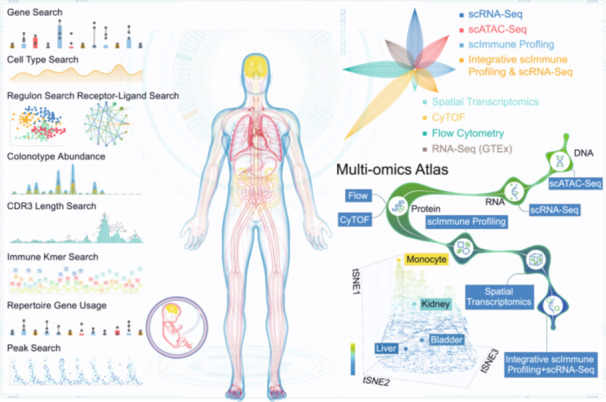
Overview of the single cell analyst database. Key features of the single cell analyst database, covering multi‐omics phenotypic features of over 100 adult and fetal tissues. RNA‐seq (GTEx), RNA sequencing from genotype‐tissue expression project; tSNE, t‐distributed stochastic neighbor embedding; CDR3, complementarity‐determining region 3.

Considering these limitations, we developed single‐cell analyst (www.singlecellanalyst.org), a web‐based platform offering streamlined workflows for six single‐cell omics types, i.e., single‐cell RNA sequencing (scRNA‐seq), single‐cell assay for transposase‐accessible chromatin using sequencing (scATAC‐seq) [9], scImmune profiling (single cell immune profiling) [10], single‐cell copy number variations (scCNV) [11], cytometry by time‐of‐flight (CyTOF) [12, 13], flow cytometry [14, 15], and spatial transcriptomics [16] (Figure 1). Complementing its extensive omics coverage, single cell analyst incorporates in addition over 20 analysis tools for stepwise data analysis and visualizations, further increasing its analysis flexibility. This protocol builds upon our previously published work, the single cell atlas (Figure 2) and the human transcriptome cell atlas, covering over a hundred tissue types in both human adults and fetuses [17, 18].

This platform integrates advanced algorithms, interactive visualizations, and intuitive interfaces, enabling researchers to conduct comprehensive analyses without programming skills. Here, we describe the capabilities of the platform, technical framework, benchmarking results, and case studies demonstrating its utility.

## RESULTS

Single‐cell analyst is an inaugural web server that is both accessible online and available for download, which incorporates six unique single‐cell omics types, encompassing scRNA‐seq [4], scATAC‐seq [9], scImmune Profiling [10], scCNV [11], CyTOF [12, 13], and flow cytometry [14, 15]; as well as spatial transcriptomics [16], covering almost all cutting‐edge single‐cell omics technologies (Figure 1). Single‐cell analyst goes beyond merely accommodating a broad array of omics types; it stands as an intuitive, all‐inclusive platform wherein every omics workflow includes components of quality control, data processing, an array of dynamic phenotypic assessments, as well as interactive visualization outputs (Figure 1 and Table S1). In the design of all analytical frameworks, we prioritized simplicity and uniformity in the data submission procedure across all omics analysis tools to reduce the complexity of the user‐interface (UI) webserver given the extensive analytic steps. The server is complemented by detailed vignettes for each omics type and demonstration videos, aiding users in understanding and navigating the workflows. For an online webserver, the offered random‐access memory (RAM) for each workflow or standalone tool is 8GB, and the downloadable version of the web server is dependent on the local server environment deployed by the user.

### Equipment

Hardware

- Using the online web‐server version of single cell analyst: Chrome browser is preferred. Any Windows/Linux/Mac OSX computers with at least 8GB of RAM and 4 cores of CPU.
- Using the offline docker version of single cell analyst: Windows/Linux/Mac OSX computers with at least 16GB of RAM and 8 cores of CPUs.

Software

- Using the online web‐server version of single cell analyst:
  1. Any version of the Chrome browser (https://www.google.com/chrome/) after the year 2022, to serve as a platform for running the online version of the single cell analyst.

- Using the offline docker version of single cell analyst:
  2. Docker software (https://docs.docker.com/desktop/), is a platform for running the offline version of the single cell analyst.
  3. Single‐cell analyst docker images (https://github.com/singlecellanalyst?tab=packages), a repository containing single‐cell analyst individual omics workflow package.

Software setup

Download and install the software

- Using the online web‐server version of single cell analyst: download and install the chrome browser to the computer meeting the minimum hardware requirement as mentioned above.
- Using the offline docker version of single cell analyst:
  1. Download and install the docker software to the computer with hardware meeting the requirement. Follow the step‐by‐step installation guide within the docker software you have downloaded. In the settings of the installed docker software, set RAM and CPU core to the minimum requirement for hardware as stated above.
  2. Open the pre‐built command line software in your computer (for Mac OSX and a Linux system, it is the terminal software; for Windows, it is the command prompt).
  3. Download the single‐cell analyst docker images that suit your data omics types by entering the following commands in the command line software. The following docker command enables you to download different omics workflow docker images from the single cell analyst GitHub repository:
    - scRNA‐seq data:
      docker pull ghcr. io/singlecellanalyst/scascrna:main
    - scATAC‐seq data:
      docker pull ghcr. io/singlecellanalyst/scascatac:main
    - scImmune profiling data:
      docker pull ghcr. io/singlecellanalyst/scascimmune:main
    - scCNV data:
      docker pull ghcr. io/singlecellanalyst/scasccnv:main
    - Spatial transcriptomics data:
      docker pull ghcr. io/singlecellanalyst/scaspatial:main
    - CyTOF data:
      docker pull ghcr. io/singlecellanalyst/scacytof:main
    - Flow cytometry data:

docker pull ghcr. io/singlecellanalyst/scaflow:main

Running the single‐cell analyst

- Using the online web‐server version of single cell analyst:
  1. Go to the analysis platform of the single‐cell analyst web portal: https://www.singlecellanalyst.org/analysis.
  2. Enter project information and upload the files of your omics type (refer to the instructions for each omics data type under the vignette tab: https://www.singlecellanalyst.org/vignettes).
  3. Click run to start the automated analysis workflow.
  4. Waiting time varies depending on the size of your data set. For a project with four samples of scRNA‐seq data, the time to completion of the analysis is around 20 min.
  5. Refer to the list of vignettes under the vignette tab (https://www.singlecellanalyst.org/vignettes) and demonstration videos for more information on interpreting the output of the analysis.
- Using the offline docker version of single cell analyst:
  1. After downloading the docker images from the single cell analyst repository, open the docker software on your computer.
  2. Go to the image tab in the software.
  3. Click the run button under actions at the side of the docker image that you would like to run.
  4. Under the optional settings, enter 127 under the Ports section and click Run.
  5. In your browser, go to: http://localhost:127/.
  6. Enter project information and upload the files of your omics type.
  7. Click run to start the automated analysis workflow.
  8. Waiting time varies depending on the size of your data set. For a project with four samples of scRNA‐seq data, the time to completion of the analysis is around 20 min for a computer and the docker environment with 32GB of RAM and 8 cores of CPU.
  9. To interpret the output.

Timing

Waiting time varies depending on the size of your data set and for the local docker version of our platform, time taken will also depend on the RAM and CPU environments on your computer. As the entire analysis workflow only consists of one click to automate the entire workflow, there is only one step for the entire analysis that the user should taken note of for the analysis timing.

1. scRNA‐seq data: For a project with four samples, the time to completion of the analysis is around 20 min.
2. scATAC‐seq data: For a project with one sample, the time to completion of the analysis is around 10 min.
3. scImmune profiling data: For a project with four samples, the time to completion of the analysis is around 20 min.
4. scCNV data: For a project with two samples, the time to completion of the analysis is around 30 min.
5. Spatial transcriptomics data: For a project with three samples, the time to completion of the analysis is around 20 min.
6. CyTOF data: For a project with 10 samples, the time to completion of the analysis is around 20 min.
7. Flow cytometry data: For a project with 10 samples, the time to completion of the analysis is around 20 min.

Troubleshooting

Following the instructions above should prevent any issues or the need for troubleshooting during analysis. However, if you are using the online version of the Analyst platform and your data size exceeds 2GB, we recommend switching to the docker version of our platform. The online version of single cell analyst may experience out‐of‐memory errors and be unable to handle large data sizes. For the offline version, users should ensure that their computers have a high CPU and RAM capacity, specifically more than 32GB of RAM and 8 CPU cores, to manage larger files effectively.

Anticipated results

Demonstration videos and screenshots of the anticipated results can also be found under the list of vignettes under the vignette tab of our single‐cell analyst platform: https://www.singlecellanalyst.org/vignettes.

### Features of the single‐cell analyst

**Figure 3:**
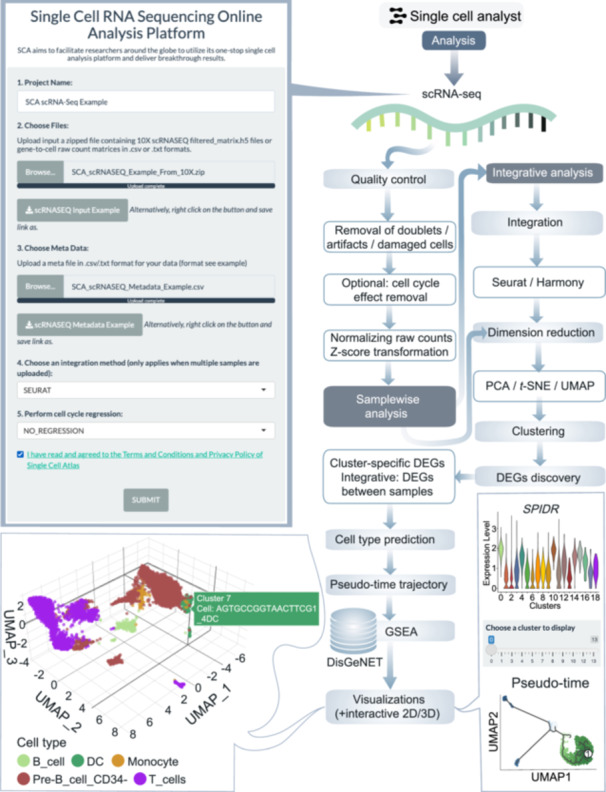
Analysis workflow for the scRNA‐seq framework. Key steps in the workflow for scRNA‐seq analysis on the multi‐omics server. DC, dendritic cells; DEGs, differentially Expressed Genes; DisGeNET, disease gene network; GSEA, gene set enrichment analysis; PCA, principal component analysis.

Within the scRNA‐seq workflow, other than quality control (QC) and data processing steps (Table S1 and Figure 1), evaluations will be made regarding cell type predictions, differential expression analysis, pathway and enrichment analyses, as well as pseudotime trajectories of clusters and cell types (Figure 3). Users will have the flexibility to designate each cluster or cell type as a starting point. In cases with multiple samples, an integrated analysis will commence to carry out comprehensive phenotypic discoveries (Figure 3).

**Figure 4:**
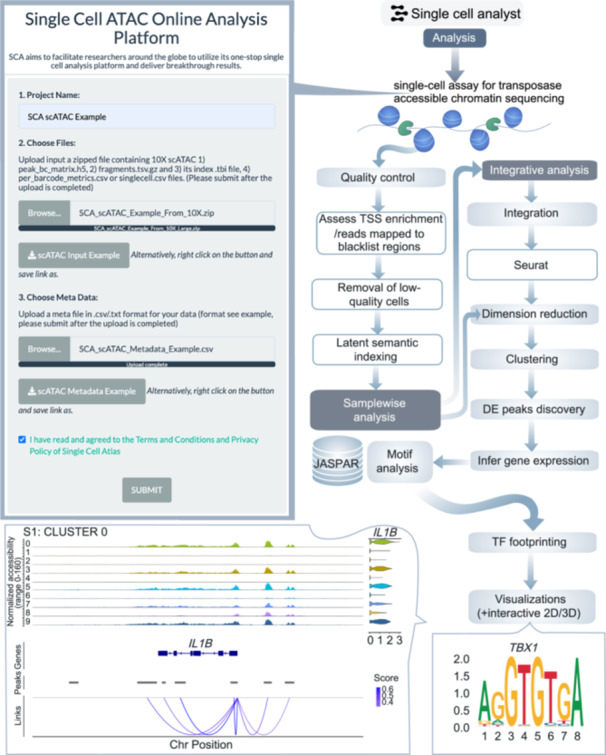
Analysis workflow for the scATAC‐seq framework. Key steps in the workflow for single‐cell assay for transposase‐accessible chromatin using sequencing (scATAC‐seq) analysis on the multi‐omics server. Chr, chromosome; DE peaks, differential accessible peaks.

Regarding scATAC‐seq, transcription start sites (TSSs) undergo scrutiny as part of the QC process. Notable peaks will be displayed for each cluster, with inferred gene activities (Table S1 and Figure 1). The process will also include motif footprinting analysis and the identification of enriched motifs. Computation of cis‐co‐accessible networks will be carried out to identify co‐accessible cis‐sites. The additional merged analysis will be carried out for multiple samples (Figure 4).

**Figure 5:**
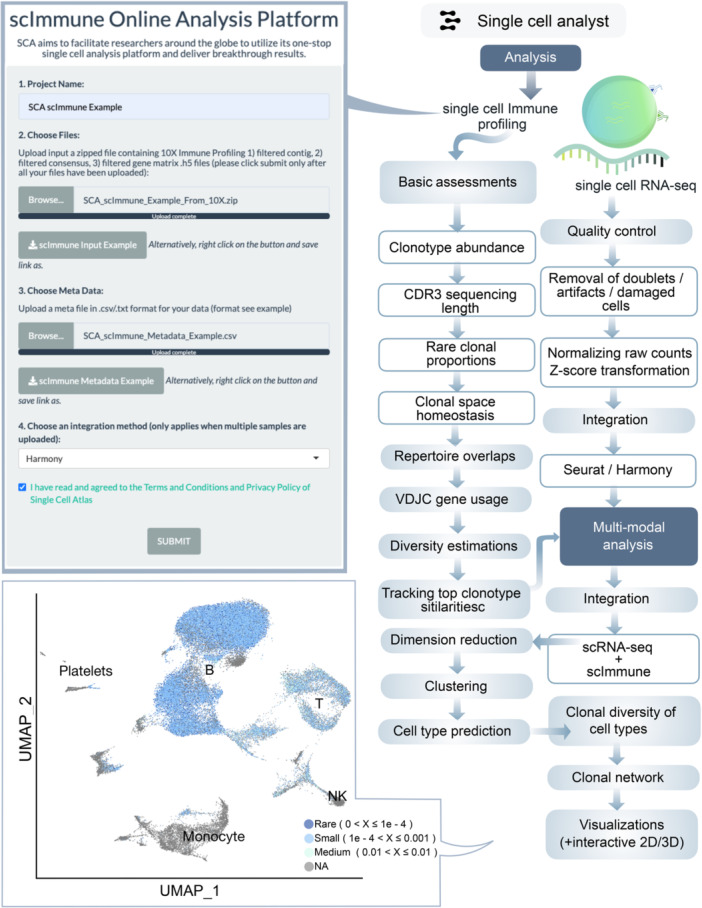
Analysis workflow for the scImmune Profiling framework. Key steps in the workflow for scImmune Profiling analysis on the multi‐omics server. NK, natural killer cells; VDJC, variable, diversity, joining, and constant gene regions.

In the realm of scImmune Profiling, the quality control assessments (QC assessments) incorporate elements such as clonotype abundance and the distribution of Complementarity‐Determining Region 3 (*CDR3*) sequencing lengths. The analytic process will extend to repertoire overlap exploration between samples, spectratyping, computation of VDJC gene usage, clonal diversity estimations, top repertoire discoveries, analysis of amino acid k‐mer sequence motifs, and clonotype homeostasis assessment (Table S1 and Figure 1). If a set of multimodal 10 × 5' scImmune profiling samples is submitted, an integrative multi‐omics analysis will be initiated. The mapping of repertoires onto their corresponding scRNA‐seq data will assess clonal expansions for inferred cell types (Figure 5).

**Figure 6:**
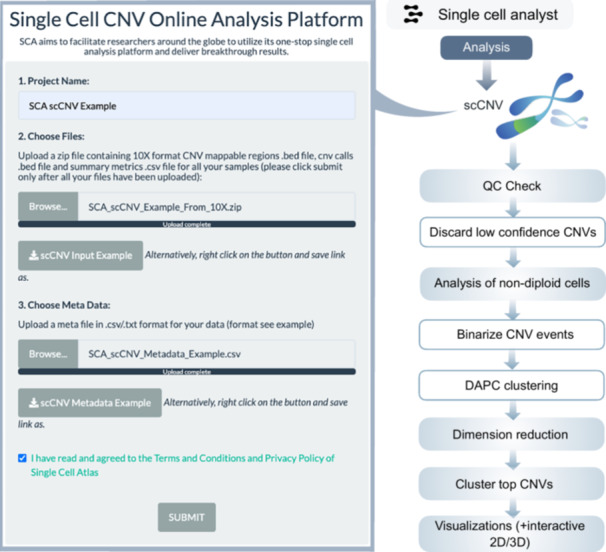
Analysis workflow for the single‐cell copy number variations (scCNV) framework. Key steps in the workflow for scCNV analysis on the multi‐omics server. DAPC, discriminant analysis of principal components.

In relation to scCNV, the process includes ploidy assessment and determination of prominent copy number variations (CNV) events in each cluster (Table S1 and Figure 1). In the process of each sample analysis, bed files and cell identities from summary metrics files are cross‐verified. CNV positions are assigned differential ploidy levels, and noisy cells are identified. Selected CNV events are used for the hierarchical clustering of cells. Further analysis is conducted on non‐diploidy cells with specific CNV events. Clustering utilizes the discriminant analysis of principal components [19] (DAPC). Using principal component analysis (PCA) components, the Bayesian information criterion (BIC) determines the number of clusters. Visualizations are created post‐clustering. The top 50 CNV events are displayed, and hierarchical clustering identifies potential CNV clusters. For multi‐sample projects, the proportion of cluster occupancy and a phylogenetic tree are assessed and depicted (Figure 6).

For spatial transcriptomics, the general analytic framework applies, with differential expression analysis (DE analysis) carried out to identify DE genes at each cluster of spots. Dimensionality reduction and clustering results will be overlaid onto their spatial images for enhanced interpretation. A multiple‐sample project submission will trigger an automatic integrative analysis. If scRNA‐seq data are provided alongside spatial transcriptomics data, an integrative multi‐omics analysis will be executed (Table S1 and Figures 1, S1).

In the context of CyTOF, raw. fcs files can be directly uploaded to the analytic framework. A provision of multiple samples will instigate an integrative analysis. The added analysis includes a comparison of the expression of every marker within groups, feature selection based on non‐redundancy scores, and primary and secondary dimensionality reductions. Meta clustering will be performed to assemble cells with similar phenotypes into a larger set of 100 clusters, primarily reducing one of the two data dimensions to 100. Secondary dimensionality reduction and clustering stages will be carried out based on the primarily reduced data (Table S1 and Figures 1, S2).

For flow cytometry, single‐cell events will be automatically gated from the provided raw. fcs flow files, with any debris or doublets discarded. Further QC will include sample distance analysis to evaluate inter‐and intra‐group variabilities and detect possible outliers. Marker importance will be evaluated using non‐redundancy scores (Table S1 and Figures 1, S3).

### Utility in addressing biological questions

Single‐cell analyst enables a wide array of downstream analyses tailored to answer diverse biological questions across different omics types. For example, in scRNA‐seq workflows, clustering results allow users to identify distinct cell populations, while differential expression analysis highlights genes associated with specific cell types or experimental conditions. Enrichment analysis links these genes to pathways and diseases, providing insights into functional mechanisms. Pseudotime trajectory analysis can be applied to study cell differentiation or dynamic processes, such as immune cell activation. By visualizing these results through publication‐ready plots and interactive tools, researchers can readily interpret their data to answer biological questions, such as uncovering tumor microenvironment heterogeneity or identifying biomarkers of disease progression.

#### scRNA‐seq analysis

Within the scRNA‐seq workflow, beyond the standard QC and data processing steps, single‐cell analyst enables users to evaluate cell type predictions, perform differential expression analysis, conduct pathway and enrichment analyses, and infer pseudotime trajectories for clusters or cell types. Users can specify starting points for pseudotime analysis based on their biological questions. For multi‐sample datasets, the platform conducts integrated analysis, allowing users to compare phenotypic differences between healthy and diseased samples or other experimental conditions. This workflow is ideal for investigating gene expression dynamics and cell state transitions in various biological contexts.

#### scATAC‐seq analysis

In the scATAC‐seq workflow, QC steps such as TSS enrichment and nucleosome banding pattern assessment are complemented by downstream analyses, including the identification of accessible chromatin regions and motif footprinting. The workflow also calculates gene activity scores, enabling researchers to link chromatin accessibility to gene expression. Users can explore cis‐co‐accessible networks and enriched motifs, providing insights into regulatory elements driving cellular functions. For multi‐sample datasets, integrated analyses reveal shared and unique chromatin features across conditions.

#### scImmune profiling

The scImmune Profiling workflow is tailored for immune repertoire analyses, offering detailed insights into clonotype abundance, *CDR3* sequence length distributions, and VDJC gene usage. The platform facilitates repertoire overlap analyses, spectratyping, and clonal diversity estimations. For multimodal datasets, the integration of scRNA‐seq and immune profiling data allows the mapping of clonotype expansions onto predicted cell types. This approach enables researchers to study immune responses in health and disease, such as clonal expansions in cancer immunotherapy or autoimmunity.

#### scCNV analysis

The scCNV workflow provides a detailed view of CNVs at the single‐cell level. It identifies ploidy levels and clusters cells based on CNV profiles, facilitating the detection of subpopulations with specific genomic aberrations. For multi‐sample datasets, phylogenetic analyses and hierarchical clustering enable the identification of shared and unique CNV patterns across samples. This workflow is particularly suited for studies of cancer, where CNVs play a critical role in tumor progression and heterogeneity.

#### CyTOF analysis

The CyTOF workflow processes raw. fcs files to identify cellular phenotypes through dimensionality reduction and clustering. Users can explore marker expression across clusters, perform feature selection, and assess the proportions of samples contributing to each cluster. The workflow includes meta‐clustering, which groups similar phenotypes into larger clusters, aiding in the identification of rare cell types or states. This approach is particularly useful for studying immune cell heterogeneity in complex diseases or treatment responses.

#### Flow cytometry

The flow cytometry workflow automates gating strategies to exclude debris and doublets, followed by dimensionality reduction and clustering. Marker expression is assessed across clusters, with non‐redundancy scores identifying the most informative markers. Integrated analyses across samples provide insights into population‐level variations and intergroup differences. This workflow is ideal for immune monitoring and biomarker discovery, where precise phenotyping is crucial.

#### Spatial transcriptomics

In the spatial transcriptomics workflow, users can visualize gene expression patterns directly on tissue images, linking spatial localization to molecular profiles. Dimensionality reduction and clustering results are overlaid onto spatial images to provide a detailed view of tissue organization. For multi‐sample datasets, integrative analyses enable comparative studies of tissue architecture under different experimental conditions. When paired with scRNA‐seq data, the workflow performs multimodal integration, providing a holistic view of spatially resolved gene expression.

#### Webserver standalone tools and other features

Standalone functional tools comprise a broad array of analytic tools and plotting choices for common or omics‐specific datasets (Figure S2), with user‐adjustable features to enhance their practical applicability. Analytic tools include independent QC, dimension reduction, clustering tools, automated cell type annotation steps, etc., while others include the visualization plots of their single‐cell omics output data or raw input data, such as CyTOF/flow cytometry flow cytometry standard (FCS) file channel visualizations.

The forum page on the platform will enable user interaction, suggestions, and queries, thus allowing SCA to continuously improve, update, and provide new features for its users. This interactive approach aims to foster broader scientific community involvement in all facets of single‐cell omics.

### Comparison with other methods

In assessing the capabilities of single cell analyst against other popular webservers, we found that none of them matched the breadth of analysis steps, omics types (Table S1), or offered the customization of standalone analysis (i.e., the option to skip sequential analyses and directly advance to a specific step of interest) provided by single‐cell analyst. As illustrated in Table S1, while many webservers provide both online and offline analysis capabilities, their scope remains relatively narrow. Web servers such as Automated Single‐cellAanalysis Portal (ASAP) [20], SingleCAnalyzer [21], Analytical Single‐cell seurat‐based Web Application (Asc‐seurat) [22], Alona [23], Single Cell Toolkit (SCTK) [24], Nucleic Acid SeQuence Analysis Resource (NASQAR) [25], ICARUS [7] provide analytic workflows exclusively for scRNA‐seq technologies, with minor variations in quality control, data processing, and phenotypic assessment steps (Table S1). Other than that, analytic platforms including Biological Interpretation Of Multi‐omics EXperiments (BIOMEX) [26] and Single‐Cell Integrative Analysis Platform (SCIAp) [3] broaden their offering to include two types of single‐cell omics types. On the other hand, ezSingleCell [5] and Cellar [6] expand further by accommodating data from three omics types, namely scRNA‐seq, scATAC‐seq, and spatial transcriptomics. However, none of the aforementioned webservers come close to the comprehensive scope of single‐cell analyst, which remains the only platform capable of delivering a diverse suite of analytic workflows covering six single‐cell omics types and spatial transcriptomics, accommodating the outputs from different technologies of these omics types (Table S1). Further differentiating single‐cell analyst from many existing tools is its broad array of standalone tools. This offers the unique advantage of independent analysis steps, eliminating the need for a sequential progression through a series of analyses before reaching the step of interest. In essence, this means that the analysis can commence from intermediate steps, enhancing the overall flexibility of the tool. Single‐cell analyst sets itself apart with a unique feature that none of these webservers offer, which is its extensive connectivity to its database, the single cell atlas (SCA) [17], www.singlecellatlas.org, comprising five single‐cell omics, spatial transcriptomics, and bulk RNA‐seq database sections (Figure 2). This database encompasses normal samples from over 90 distinct adult and fetal tissues. This comprehensive resource establishes a solid foundation for comparative analysis, allowing users to deeply explore their data in relation to a wide range of tissue types.

### Expertize needed to implement the protocol

All analyses will be conducted on a web‐based platform featuring a user‐friendly interface with simple, step‐by‐step navigation. While no coding skills are necessary, users should possess a fundamental understanding of interpreting omics data results.

The workflow necessitates users to upload their data to our web‐based portal for analysis. Consequently, projects with numerous samples or large file sizes might experience prolonged upload times, and a stable internet connection is essential for uninterrupted analysis. To mitigate this, users handling large files can use the docker version of the analysis platform, which allows offline operation without needing a stable internet connection. The duration of such analyses varies based on the local user environment. For the docker version, refer to the code availability section.

### Execution and scalability

#### Scalability and performancettesting

We tested the seven omics workflows (scRNA‐seq, scATAC‐seq, immune profiling, scCNV, CyTOF, flow cytometry, and spatial transcriptomics) in the single cell analyst using the provided example datasets available within the app. For larger datasets, docker deployment on servers or high‐performance machines is strongly recommended.

#### Performance results

Performance metrics, including runtime, were assessed across various CPU and RAM configurations. Table S2 shows the results and recommendations for each workflow.

- Online version: Best suited for datasets less than 2GB in size as the online platform is hosted on a server with 8GB RAM and with a flexible number of CPUs provided.
- Docker version: Strongly recommended for datasets exceeding 2GB. For larger datasets, using the docker version on systems with adequate memory and CPUs are strongly recommended. Users should run the platform on a server or high‐performance local machine configured according to Table S3.

## DISCUSSION

The extensive functionalities and user‐friendly design of the platform position single‐cell analyst as a valuable resource for advancing clinical research and diagnostics. For instance, the platform enables the identification of disease biomarkers across omics types, elucidation of disease heterogeneity, and exploration of mechanisms underlying therapy resistance. In cancer immunotherapy, for instance, single‐cell analyst can identify molecular signatures distinguishing responders from non‐responders, aiding in the development of more effective treatment regimens. Additionally, visualization tools enable the generation of publication‐ready figures and clinical‐grade reports, facilitating communication between researchers and clinicians. By streamlining complex single‐cell analyses, single‐cell analyst contributes to the development of precision medicine and accelerates the application of single‐cell technologies in clinical diagnostics and therapeutic discovery.

To maintain dynamism and responsiveness, we manage version control of single‐cell analyst on GitHub (https://github.com/singlecellanalyst/SCAWebserver), where we address issues, make improvements, and incorporate new features. To cater to user needs, we have dockerized all workflows, enabling users to run analyses locally or on their web servers. Details and applications of all omics workflows can be retrieved and downloaded from https://github.com/singlecellanalyst?tab=packages. Our commitment extends to keeping our platform up‐to‐date with the latest advancements in single‐cell analyses, ensuring that single‐cell analyst remains a premier tool in this rapidly evolving field. We acknowledge the existence of numerous remarkable tools, each with unique strengths. Nonetheless, we firmly believe that the comprehensive nature of single‐cell analyst, combined with our commitment to continual enhancement of its functionalities, carves a niche for it as a valuable and user‐friendly single‐cell multi‐omics analysis platform.

To address potential technical issues, single cell analyst provides multiple support mechanisms. Users can access the Forum tab (https://www.singlecellanalyst.org/forum) or the contact tab (https://www.singlecellanalyst.org/contact) on the platform for troubleshooting and discussions. Additionally, the GitHub repository serves as a space for issue tracking and resolution. Built‐in error handling mechanisms in the platform will notify users of input or format errors and will provide prompts for corrective actions, ensuring seamless user experience and analytical workflows.

Currently, single‐cell analyst is designed primarily for human data analysis, and we will further enhance our platform in future releases to support organisms other than humans. In addition, future enhancements of the platform will also focus on the importance of joint analyses for multimodal single‐cell datasets, such as scATAC‐seq and scRNA‐seq from identical cells. However, implementing such functionality requires significant computational resources, which is currently limited in the online version due to an 8GB memory restriction. While the docker version could technically support such analyses, we aim to maintain alignment between the online and docker versions in this initial release to ensure consistency. In future updates, we plan to expand the capabilities of the docker version to include joint analyses, thereby addressing this limitation. Besides, we will also be expanding support for additional omics data types, such as metabolomics, to facilitate more comprehensive multi‐omics analyses. To strengthen the integrative analysis capabilities of the platform, we aim to implement more robust pipelines for the integration of diverse data modalities. Advances in predictive modeling using machine learning methods will be integrated into the platform to provide analysis for prognostic and diagnostic purposes. To meet the diverse needs of researchers, we plan to introduce enhanced customization features, enabling users to tailor analytical workflows and visualizations. Efforts will also be directed at improving computational scalability to ensure smooth performance with increasingly larger datasets, accommodating the demands of high‐throughput single‐cell studies. The platform will continue to evolve through community‐driven development, leveraging feedback and feature requests from users via the forum and GitHub repository. These advancements will ensure that single‐cell analyst remains a state‐of‐the‐art, accessible resource for the global single‐cell research community.

## CONCLUSION

In our pursuit of fostering accessibility and efficiency in single‐cell omics research, we developed single‐cell analyst, a comprehensive multi‐omics analytic platform that supports six single‐cell omics types and spatial transcriptomics. The platform encapsulates popular single‐cell omics workflows, providing a significant boon to researchers without a substantial computational background. By eliminating the need for dependency downloads and circumventing the complexities often associated with software installations, single‐cell analyst addresses critical barriers to entry, enabling broader adoption of single‐cell technologies. single cell analyst excels in its efficiency, user‐friendliness, and broad scope, making it a formidable resource in the single‐cell analysis landscape. By streamlining workflows, automating key steps, and offering interactive, publication‐ready visualizations, it opens avenues for bench scientists to delve into multi‐omics analysis, delivering advantages to a broad spectrum of the research community. Its coding‐free design and integrative analysis capabilities lower the technical threshold, empowering researchers from diverse backgrounds to explore and interpret complex single‐cell data with ease. The ability of the platform to analyses datasets across different omics fosters a systems‐level understanding of biological phenomena, offering unprecedented flexibility for multi‐omics analyses. This capacity accelerates discovery processes and empowers researchers to generate insights into cellular heterogeneity, tissue architecture, and molecular mechanisms. By paving the way for innovative approaches to complex questions in biology and medicine, single‐cell analyst not only drives the adoption of single‐cell technologies but also broadens their application and facilitates interdisciplinary collaboration, representing a transformative advancement in the field of single‐cell research.

## METHODS

### Platform overview

Single‐cell analyst is a dual‐access platform supporting online and offline (docker) workflows. The platform is implemented using R (v4.2.0) and its shiny applications for front‐end and back‐end integration. Each workflow is implemented with established bioinformatics tools and libraries, ensuring compatibility with standard file formats and technologies. A complete list of dependencies, R packages, versions, and links is provided in Table S4. Key dependencies include seurat (v4.1.0) [27], Monocle3 (v1.0.0) [28, 29, 30], Harmony (v0.1.0) for scRNA‐seq; Signac (v1.6.0) [31] for scATAC‐seq; scRepertoire (v1.3.5) [32] for scImmune Profiling analysis; etc. (Tables S5, S6).

## INPUT FORMATS AND PRE‐PROCESSING

Input requirements:

1. scRNA‐seq: Gene‐cell count matrices in. csv or. txt format, or 10X Genomics post‐quantification outputs (.h5).
2. scATAC‐seq: Fragment files (.bed), peak annotations, and cell‐by‐peak matrices.
3. scImmune Profiling: Filtered clonotype matrices and *CDR3* sequences (.csv) from 10X Genomics, including its corresponding scRNA‐seq data.
4. Spatial Transcriptomics: Expression matrices with spatial coordinates (.h5 or.csv).
5. scCNV: Bed files containing copy number variations and summary metrics.
6. CyTOF/flow cytometry: Raw.fcs files.

Pre‐processing steps:

1. scRNA‐seq: QC filtering (cells with >200 genes, <5% mitochondrial reads), log‐normalization, and scaling using NormalizeData and ScaleData functions in seurat (Figure 3).
2. scATAC‐seq: Nucleosome banding pattern, TSS enrichment (TSSEnrichment in Signac), and blacklist region removal (Figure 4).
3. scImmune profiling: Clonotype filtering (≥2 reads per cell), VDJC annotation (Figure 5).
4. scCNV: Discard low confidence CNVs (event confidence <100) (Figure 6).
5. Spatial transcriptomics: Normalization using SCTransform (regularized negative binomial) in seurat (Figure S1).
6. CyTOF: Arcsine transformation with a cofactor of 5 for scaling raw marker expression (Figure S1).
7. Flow cytometry: Arcsine transformation with a cofactor of 150 for scaling raw marker expression (Figure S1).

### R packages and dependencies

Table S4 summarizes the R packages, versions, and their URLs used for each omics type.

A comprehensive list of dependencies for each workflow is provided in Table S3.

### Algorithms and parameters for analysis

1. Dimensionality reduction:
  ∘ Algorithm: PCA, Harmony (batch correction), Uniform Manifold Approximation and Projection (UMAP), and t‐distributed Stochastic Neighbor Embedding (t‐SNE).
  ∘ Parameters: PCA components (30 by default), UMAP min_dist = 0.3, n_neighbors = 15.
  ∘ Dependency: Harmony (v0.1.0) for batch correction.
2. Clustering:
  ∘ Algorithm: k‐nearest neighbor (k‐NN), shared nearest neighbor (SNN) modularity optimization.
  ∘ Parameters: Resolution = 0.8 (FindClusters function in seurat).
  ∘ Dependency: seurat (v4.1.0).
3. Differential expression (DE) analysis:
  ∘ Algorithm: Wilcoxon rank‐sum test (seurat), logistic regression for scATAC‐seq.
  ∘ Thresholds: Adjusted *p‐*value <0.05, log fold‐change >0.25.
4. Pathway enrichment:
  ∘ Algorithm: GSEA (fgsea v1.22.0), DisGeNET enrichment analysis (DOSE v3.20).
  ∘ Parameters: Top 500 differentially expressed genes (DEGs) per cluster, adjusted *p‐*value < 0.05.
  ∘ Dependency: ClusterProfiler (v4.2.2).
5. Trajectory analysis:
  ∘ Algorithm: Monocle3 (v1.0.0), principal graph inference using reversed graph embedding.
  ∘ Parameters: Start nodes are cluster‐defined, minimum branch size = 10.
6. Peak calling and gene activity (scATAC‐seq):
  ∘ Algorithm: TF‐IDF normalization, motif footprinting using Signac.
  ∘ Parameters: TSS enrichment > 2, peak width = 200 bp.
  ∘ Dependency: JASPAR2020 database.
7. Integration:
  ∘ Algorithm: seurat reciprocal principal component analysis (seurat reciprocal PCA), Harmony integration.
  ∘ Parameters: Features = 2000, integration anchors = 30.
8. CyTOF and flow cytometry:
  ∘ Algorithm:Flow cytometry self‐organizing map (FlowSOM) (v2.2.0), Consensus Clustering.
  ∘ Parameters: Meta clusters = 100, final clusters optimized using Elbow method

## SOFTWARE AND PACKAGE VERSIONS

1. R Libraries:
  ∘ seurat (v4.1.0), Monocle3 (v1. 0.0), Signac (v1.6.0), ClusterProfiler (v4.2.2).
2. Docker: Docker desktop (v4.12.0) for offline execution.

### Analysis framework

The platform supports streamlined workflows for analyzing raw post‐quantification files. These workflows include automated pipelines to generate post‐analysis results with interactive visualizations. A single‐click submission after uploading the necessary input data and sample sheet triggers downstream analyses (Figures 3, 4, 5, 6, Figures S3–5). Below, we detail each omics‐specific workflow.

### scRNA‐seq analysis framework

The analysis framework is designed for post‐quantification scRNA‐seq data from 10X Genomics. Direct output from their post‐quantification data will be used as input to the framework. The analysis also supports input from other technologies with gene‐cell raw matrices in. csv or. txt format (Figure 5). The analysis supports multiple scRNA‐seq sample submissions for integrative analysis and has functions from popular packages such as seurat and monocle3 [28, 29, 30].

#### Quality control checks

For each sample in a submitted project, a series of QC metrics will be calculated before QC filtering. The percentage of mitochondrial genes expressed in each cell will be computed for the depiction of damaged cells or artifacts present in each sample. The number of genes and RNA molecules detected per cell will be calculated simultaneously. To observe any cell cycle effect present in a sample, the sample data will be normalized and scaled using highly variable genes present in the sample. PCA and UMAP will be run based on (1) a list of cell cycle markers (S and G2M phase markers) and (2) highly variable genes to compare and contrast the effect of cell cycle markers on the data. If there is an obvious cell cycle heterogeneity observed in the samples, depending on the biological question of the project, the user could choose to rerun the project with cell cycle regression selected (if not selected) if the cell cycle effect should be removed. Two options are available to the user, including two phases and phase difference cell cycle regressions. The former will mitigate the effect introduced by all cell cycle phases, and the latter will remove only the difference between G2M and S phases. The pre‐filtering results will be visualized and presented to the user.

#### Quality control check after filtering, dimension reduction, and clustering proceeding

Filtering is then carried out for each sample; cells with the number of genes expressed between 200 (exclusive) and 25,000 (inclusive), and less than 5% of mitochondrial genes expressed (exclusive) are retained. This step removes possible empty droplets, doublets, artifacts, and damaged cells present in the sample as a result of experimental procedures. Sample data will be normalized based on the filtered data and scaled using highly variable genes. Depending on the choice of the user to perform cell cycle regression, the cell cycle will be performed if chosen, and the effect of the cell cycle on the data will be removed, depending on the regression choice. The same set of QC metrics will be displayed for the user. For each sample, PCA, UMAP, and t‐SNE projections will be calculated using the highly variable genes in the filtered data. k‐NN and SNN will be computed based on the first 30 PCA dimensions, and clustering will be done using the *FindClusters* function in seurat with a resolution of 0.8. Clustering results will be shown in both static 2D and 3D interactive UMAP and t‐SNE plots.

#### DE analysis based on clustering results

Differential expression analysis will be performed for each cluster and each gene using the Wilcoxon rank‐sum test in seurat. A threshold is set for each comparison to require the peak to be detected at a minimum of 0.1 in proportion in either of the two groups of cells. The final list of DEGs for each cluster is retained by a Bonferroni‐corrected threshold of adjusted *p* < 0.05 and >0.25 of absolute average log‐fold change of the gene in the cells of the cluster compared to cells present in other clusters. The median expression of the DEGs in the log‐normalized data of the sample will be computed and represented in a heatmap visualization. The top gene density distributions will be plotted. Post‐dimension reduction and clustering results will be represented in UMAP, t‐SNE, and PCA visualizations. DEGs for each cluster of each sample will be displayed in a table so that the user can use the DEGs for cluster cell‐type annotation. To aid cell‐type annotation, the automated cell‐type annotation will be carried out using SingleR [33] based on the clustering result, and human primary cell atlas is used as the annotation reference [33]. To perform automated cell type annotation, SingleR compares the gene expression profile of each cell to the reference expression data set, and the human primary cell atlas, which contains profiles for various cell types in the human body. This comparison uses a correlation‐based approach, assigning the most likely cell type label based on the highest similarity to reference profiles. Please note that automated annotation is provided for self‐reference, and manual annotation based on DEGs is encouraged, as the accuracy of annotation depends heavily on the reference data set, thus rare or tissue‐specific cell types absent in the reference may lead to misannotation. Therefore, manual validation of annotations using differentially expressed genes and biological context is strongly encouraged to ensure robust conclusions. To assist manual annotation, the median expression of the top DEGs in each annotated cell type will be computed and presented to the user in terms of a heatmap for users to validate automated cell type annotation. Annotated results will be represented in both static 2D as well as 3D interactive UMAP and t‐SNE plots.

#### Enrichment analysis

For each cluster of each sample, 500 top DEGs, ranked by decreasing average log‐fold change, will be used for enrichment analysis of disease‐gene associations based on DisGeNET [34] using DOSE [35]. Enrichment disease terms below *adjusted p* < 0.05 and Q < 0.05 will be retained, and for each cluster in the sample, the results will be displayed in terms of bar plot, dot plot, and heatmap, catering to different visualization needs. Linkages between genes and enrichment terms will be constructed with a gene‐concept network, and the results will be displayed in network graphs for each cluster and sample. The enrichment network of the disease terms will also be shown. The disease terms will be compared across clusters within each sample, and a network of disease terms clustered by clusters will be displayed. An upset plot to visualize the association of DEGs and the enrichment terms will be displayed. GSEA is also performed on the DEGs of each cluster, and the results are presented in an enrichment plot [35, 36, 37]. Cell‐type labels will be mapped onto t‐SNE and UMAP visualizations and presented to the user.

#### Pseudotime trajectory analysis

Monocle3 will be used for pseudotime analysis. The principle graph is first learned based on sample UMAP, and to maximize flexibility in the result, each cluster will be used as a starting node for pseudotime computation, and each of these results will be returned to the user. This allows the user to choose the pseudotime trajectory result based on their biological questions. The results will be displayed interactively. Alternatively, pseudotime computed based on ranked first embeddings of PCA, UMAP, and t‐SNE will also be displayed in terms of clustering results and annotated cell types.

#### Integrative analysis

For a project with multiple samples submitted, integration of samples will be carried out. An example of such a project will be a comparison between healthy and diseased samples. Group information should be provided in the metadata submitted. Depending on the choice of the integration method, the integration will be done using seurat or Harmony. If seurat integration is selected, a more stringent integration will be performed by considering each sample as a batch. This is beneficial for projects with high intra‐batch variations. Top features that vary across samples will be selected and identified as integration features. Dimension reduction based on Reciprocal PCA will be performed to identify cell pairs from each sample that are within each other's neighborhood (i.e., mutual nearest neighbors). Low‐confidence anchors will be dropped. Based on shared neighbor overlaps between cells from each pairwise sample, outliers will be removed, and the final set of integration anchors based on each pair of samples will be determined for the integration process. Depending on the anchors, pairwise integration will be performed and will be iterative for multiple samples. The integrated data set will be scaled, and PCA embeddings for the integrated data set will be computed based on highly variable genes present in the integrated data set. The dimension reduction procedure will be done using both UMAP and *t*‐SNE. If the Harmony method is selected, the integration will be done using Harmony based on the batch information provided by the user in the metadata text file. The integration will be performed based on the pre‐computed unadjusted PCA embeddings. Cells will be assigned to clusters, and the centroid for each sample in the clusters will be computed. Correction factors will be calculated for each cluster to pull the centroid of each sample within each cluster together. The location of the cells based on the current PCA embeddings will be adjusted and corrected using the correction factor specific to each cluster. This is done interactively until convergence. In terms of time complexity, Harmony will perform at a faster pace as the sample size increases. A corrected set of Harmony embeddings based on unadjusted PCA embeddings will be returned and used for subsequent dimension reductions using UMAP and *t*‐SNE. Before and after integration, QC metrics will be presented to the user and will be represented by UMAP, *t*‐SNE, and PCA representations to serve as integration quality checks for the user.

Unsupervised clustering using k‐NN and SNN modular optimization will be performed on the integrated data using seurat with a clustering resolution of 0.8. DE analysis for each cluster of the integrated data set will be performed. Similar to sample‐level DE analysis, the Wilcoxon rank‐sum test will be performed, and genes with a Bonferroni‐corrected threshold of adjusted *p* < 0.05 and >0.25 of absolute average log‐fold change will be retained as DEGs for each cluster. The median expression of the DEGs in the log‐normalized data of the sample will be computed and represented in a heatmap visualization. Automated cell type annotation will be performed using SingleR on the integrated data set based on the clustering results. Likewise, the human primary cell atlas will be used as the annotation reference. The median expression of the top DEGs in each annotated cell type will be computed and presented for validation. Proportions of samples in each cluster will also be computed and displayed.

Group‐wise comparisons in the integrated data set. For samples with multiple groups provided, group‐wise comparison within each cluster will be performed to discover genes expressed differentially between groups within each cluster using the Wilcoxon rank‐sum test. DEGs with Bonferroni corrected *adjusted p* < 0.05 is retained. No comparison will be made for comparing groups with cell counts less than 3. The DEG expression distribution across clusters and groups will be displayed.

Pseudotime trajectory analysis on the integrated data set. Likewise, using Monocle3, the principal graph will be learned using integrated UMAP embeddings. For each cluster or annotated cell type, the cluster will be treated as a pseudotime starting point, and the corresponding pseudotime based on the starting point will be computed. As mentioned, this allows the user to choose a suitable pseudotime trajectory result based on their biological questions. The results are also displayed interactively.

### scATAC‐seq analysis framework

The analysis is designed for post‐quantification scATAC‐seq from 10X Genomics. Direct output from Cellranger will be used as input to this analysis framework. Due to the large sample size of scATAC‐seq, multimodal integrative analysis is currently not supported due to memory complexities. The user is required to state the reference genome build used in the metadata file. Since scATAC‐seq data are often larger than other omics samples, the procedure may take a longer time compared to other omics analyses (Figure 6).

#### Quality control checks before filtering

The nucleosome banding pattern will be computed and visualized using Signac, and this will be done for each cell. The transcription start site (TSS) enrichment score will be calculated to examine the enrichment level in TSS regions. The score is proportional to the quality of the experiment. The number of fragments in peaks will be used to examine cellular sequencing depth and complexities. Peak counts that are too low or high are indications of low sequencing depths or doublets. Another QC check is the identification of cells with a high proportion of reads mapped to blacklist regions. These regions, identified by ENCODE, are a list of signal‐artifact regions with erroneous signals. The ratio of reads in each cell mapped to blacklist regions compared to overall reads present in the cell will be calculated [31].

#### Quality control check after filtering, dimension reduction, and clustering proceedings

Based on the QC metrics, for each sample, cells with (1) number of fragments in peaks >3000 and <20,000; (2) nucleosome signal < 4; (3) TSS enrichment score > 2; (3) blacklist ratio < 0.05; (4) percentage of reads at the peak regions > 15% is retained. This filtering process removes cells with problematic signals, as well as low‐quality cells with low sequencing depths or doublets. Normalization will be performed thereafter using the TF‐IDF procedure, which corrects for cellular sequencing depths and, at the same time, corrects for peaks by assigning higher weights to rare peaks. Unlike scRNA‐seq, feature selection for scATAC‐seq will be carried out by taking the top 25% of peaks in the sample. Dimension reduction will be carried out using singular value decomposition (SVD) on the normalized data, using the top peaks selected. This generates a set of LSI components that are used in the subsequent dimension reduction step, where UMAP and *t*‐SNE will both be carried out to dimensionally reduce the data. The first component of the LSI often captures technical variation rather than biological variation and thus will be omitted. Second to 30th LSI components will be used. After dimension reduction, the clustering step will be carried out based on the 2nd to 30th LSI components and k‐NN followed by SNN, and modular optimization using the SLM algorithm will subsequently be performed [31].

#### DE analysis based on clustering results

Differential accessibility analysis will be performed for each cluster and each peak using logistic regression in Signac. The number of peaks will be used as a latent variable to reduce the sequencing depth effect. For each comparison, peaks that could not be detected at a minimum of 0.05 in proportion in either of the two groups of cells are discarded. The final list of DEGs for each cluster is retained by a Bonferroni‐corrected threshold of adjusted *p* < 0.05 and will be shown to the user in a table format, together with dimension reduction and clustering results in the forms of UMAP and *t*‐SNE representations.

#### Inferring gene activity from scATAC‐seq and motif analysis

To relate the promoter accessibility of each gene to its gene expression, using Signac, the number of fragments mapped to the 2 kb upstream region of every gene will be tabulated, and the number of fragments at the region will be used as an activity score for each gene in each cell. The gene activity matrix will undergo normalization, followed by scaling. Based on the clustering results in scATAC‐seq earlier on, DE analysis will be performed on every gene of each cell in the gene activity matrix by comparing the activity of each gene in the cluster of cells with all other cells outside the cluster using the Wilcoxon rank‐sum test. For each comparison, genes that could not be detected at a minimum of 0.5 in proportion in either of the two groups of cells are discarded. The final list of DEGs for each cluster is retained by a Bonferroni‐corrected threshold of adjusted *p* < 0.05 and >0.25 on average log2Fold‐Change. For each peak, GC content, accessibility, and peak length will be computed. Correlation analysis will be carried out using the gene activity of every top gene derived from the DE analysis with all peaks near the gene. The top peak, regions 10 kb up‐ and downstream, and its respective top gene in each cluster will be visualized on a coverage plot for the user. DNA sequence motif analysis will be subsequently carried out. A collection of motif positions will be first queried from the JASPAR database [31, 38] and mapped to the peaks. Motif enrichment analysis will be carried out on the DEGs to find enriched motifs in these genes, and their position‐weighted matrices will be visualized. Footprinting information for motifs mapped to positions of the top 6 DEGs will be gathered and plotted.

#### Integrative analysis

For a project with multiple samples, integration of samples will be carried out. Peaks from all samples will be combined and will undergo top peak selection similar to individual sample feature selection. This is followed by normalization using the TF‐IDF procedure, and dimension reduction will be performed to acquire SVD embeddings. Nonlinear dimension reduction in terms of UMAP and *t*‐SNE will both be carried out. The second to 30th LSI components will be used before the UMAP and *t*‐SNE procedures. The clustering step will also be carried out based on the 2nd to 30th LSI components, and k‐NN followed by SNN, and modular optimization using the SLM algorithm will subsequently be done. DE analysis will be carried out for the integrated data set to obtain the top peaks. Likewise, gene activity will be inferred in the integrated data. A coverage plot will be plotted for the top peaks, together with the top DEGs.

### scImmune profiling analysis framework

The analysis is designed for multimodal post‐quantification scImmune profiling samples from 10X Genomics. Submission of more than one multimodal sample is also supported. Both T and B repertoires could be provided for the same sample, provided that this information is fully captured in the metadata provided by the user. Filtered data files should be provided, which are the default outputs from the quantification step in Cellranger. For the analysis, clonal calls by *CDR3* nucleotide sequences, amino acid sequences, VDJ genes, and *CDR3* nucleotide sequences will all be done (Figure 5).

#### Basic statistics and clonal summary

For each repertoire of each sample, basic statistics such as the number of unique clonotypes, distribution of clonal abundance, and *CDR3* sequence length distribution will be computed and visualized. Through binning, repertoire occupancies will be assessed in various ways. First, the repertoires will be ranked by their proportions, and they will then be split into bins, with the first bin containing the top 1 to 10 clonotypes, the next bin containing the top 11 to 100 clonotypes, and so on. Given that immune repertoires are diverse in nature due to random recombination of VDJ sequences, this way of representation gives more weight to the top clonotypes in terms of the visualization process (similar to plotting the proportion in a log scale). The next step is to examine the rare clonal proportions. The top bin consists of the rare clonotypes with single cells, and the second consists of clonotypes with 2–3 cells. The concept is similar to the first way of binning the visualization process. Clonal space homeostasis will also be assessed by binning the clonotypes with different proportions into rare (0 < X ≤ 1e‐04), small (1e‐04 < X≤ 0.001), medium (0.001 < X ≤ 0.01), large (0.01 < X ≤ 0.1) and hyperexpanded (0.1 < X ≤ 1) bins/groups.

#### Shared clonotype assessments

For multiple sample projects, repertoire similarity assessments will be conducted to examine the level of clonotype sharing between samples, and this is indeed very useful for different biological aims, for example, the sharing of clonotypes between individuals of the same diseased group or in terms of follow‐up studies before and after treatment. To measure the degree of similarities between repertoires, both the Morisita overlap index and a direct comparison of the number of overlapping clonotypes will be computed and displayed.

#### Gene usage analysis and spectratyping

Repertoire genes will be assessed for the level of usage, to dissect the differential gene usage across repertoires, to observe similarities, and to examine what proportion of the combination of genes contributes to the repertoire diversities. Genes will be assessed and visualized. The correlation matrix and usage by JS divergence will also be calculated and visualized. Hierarchical clustering of the repertoires will be performed for each gene based on the gene usage using the cosine similarity procedure. For any projects with more than two samples, dimension reduction will be carried out based on gene usage, and the distance between each repertoire will be visualized on dimensionally reduced PCA and multi‐dimensional scaling (MDS) plots. JS divergence is used here to preprocess repertoire similarities. Spectratyping will also be carried out to assess the distributions of genes per sequence length. The top gene segments will be computed and visualized.

#### Repertoire diversity estimation

To examine how diverse each repertoire is, a set of diversity estimation methods will be implemented, which include the Chao1 estimator, to estimate species richness; hill numbers, which tells the effective number of species in the repertoires; true diversity, reflecting the effective number of clonotypes; Gini‐Simpson index, the probability of two clonotypes taken at random are of the same type; inverse‐Simpson index; D50, the minimum number of distinct clonotypes ≥50% of total sequenced reads; rarefaction analysis, to assess species richness from sampling via extrapolation.

#### Tracking of top clonotypes

Similar to shared clonotype assessments, top clonotypes will be compared across repertoires, and this can be useful in many ways, depending on the research questions. For example, this can be useful for group‐wise repertoire comparison or follow‐up analysis of repertoires from the same individual over time.

#### Multi‐modal integrative analysis preparation

scRNA‐seq samples of the repertoires will be read in and further filtered before the integrative analysis. Cells with <200 or >6000 genes expressed in one cell are discarded. This is fixed and catered for 10× Genomics output, as we do not support other scRNA‐seq technologies here because they have differing sequencing depths at the upper limit. Post‐filtering data are then log‐normalized and scaled using highly variable genes. PCA is run to obtain a set of linearized dimension reduction embeddings before nonlinear dimension reduction, such as UMAP and *t*‐SNE. If multiple samples are supplied, an integrative analysis will be performed for the scRNA‐seq samples. Depending on the integration method chosen by the user, seurat or Harmony integration will be carried out. The same procedure for seurat or Harmony integration will be conducted as described in the scRNA‐seq analysis. Post‐integration, scaling the data and PCA will be computed on the integrated data, followed by dimension reduction using UMAP, based on PCA embeddings. If harmony is chosen, dimension reduction using UMAP will be based on corrected Harmony embeddings. Clustering will be carried out based on the first 30 PCA or Harmony embeddings and k‐NN followed by SNN, and modular optimization using the original Louvain algorithm will subsequently be done. Automated cell annotation will be carried out to predict the cell‐type identity of each cluster.

#### Multimodal integrative analysis

scRepertoire is used in this analysis. Immune repertoires will be mapped to their respective scRNA‐seq samples based on their unique cell barcodes, clonotype calls using the standard definition of clonotype, VJDC genes, and the Complementarity‐Determining Region 3 (*CDR3*) nucleotide sequence will be subsequently mapped, and clonal space homeostasis will be assessed by grouping cells into different clonal expansion groups, as mentioned previously. Rare (0 < X ≤ 1e‐04) small (1e‐04 < X ≤ 0.001), medium (0.001 < X ≤ 0.01), large (0.01 < X ≤ 0.1), and hyperexpanded (0.1 < X ≤ 1) groups. This information will be visualized in the scRNA‐seq dimensionally reduced UMAP embeddings. The proportion of clonal expansion groups is calculated for each predicted cell type to assess the proportion of expansion in each cell type group. If group information is provided in the metadata by the user (e.g., sample one is diseased, sample two controls, and so on), then the proportion will also be visualized separately between groups. Clonal diversity will also be assessed for each predicted cell type. To visualize the density of clonal expansion on the UMAP 2D space, density in the form of contours will be plotted onto the UMAP representation. To assess the network interaction of clonotypes shared between cell types, a clonal network is computed and reflected onto UMAP embeddings.

### scCNV analysis framework

The analysis is performed for multimodal post‐quantification scCNV samples from 10X Genomics. Multi‐samples integrative analysis are supported. Since CNV data are often larger than other omics samples, the procedure may take a longer time compared to other omics analyses. The analysis workflow is largely based on the 10X Genomics scCNV application demonstration notebook (Figure 6).

#### QC metrics and data processing

For each sample, bed files containing copy number calls and mappable regions of the genome will be read in and validated with the cell identities present in the summary metrics files. At each CNV position, copy number information will be binned into differential ploidy levels, from 0 to 9 in 10 separate bins, and in addition, any ploidy level above or equal to 10 will be placed into the same bin. Noisy cells will be labeled. Noisy cells are cells identified by Cellranger with uncertainty in their ploidy estimate and differing read count profiles compared to other cells. CNV events with event confidence above 100 in diploid cells will be retained, and CNV events with event confidence above 50 in non‐diploid cells will be retained. Based on the CNV profiles of each cell in the sample, hierarchical clustering will be carried out to group cells with similar CNV events together. The clustered CNV profiles will be visualized and shown to the user. The mean ploidy of each cell in each sample, based on their copy numbers in each CNV event, will be computed and visualized.

#### Analysis of non‐diploid cells

Non‐diploidy cells will be selected for downstream analysis. Non‐diploid cells with CNV event sizes in mappable regions >2000 kb with confidence >15 is retained. CNV events present in less than 5% of all cells in the sample will be removed. The final set of CNV events will be binarized to the presence of CNV events and undergo clustering using the DAPC [19]. Successive K‐means will be run using dimension‐reduced PCA components, the goodness of fit measure using the BIC will be computed, and based on the BIC information, the number of clusters will be automatically determined. UMAP is performed on the binarized CNV events, and post‐dimension reduction and clustering results are visualized. Hierarchical clustering will be carried out on the binarized non‐diploid matrix, to observe any obvious CNV clusters in each cell cluster. The top 50 CNV events in the non‐diploid cells will be selected and visualized for their CNV ploidy information at each chromosome position and will be clustered based on their clustering information. For a multiple samples project, the proportion of cluster occupancy of the non‐diploid cells for each sample will be assessed and plotted, and a phylogenetic tree will be plotted by comparing and clustering the clusters based on their median copy number.

### CyTOF analysis framework

The analysis is catered to running mass cytometry CyTOF*. fcs* files. Submission of multiple samples is supported. After doublet removal, filtered*. fcs* files will be desired as input files for the analysis (Figure S4).

#### QC and data processing

FCS format files will be read in, and scaling will be performed by arcsine transformation on the expression matrix of each sample with a co‐factor of 5. If batch information is provided, batch removal will be carried out to regress the batch variable using linear regression. The summary of cell counts in each sample will be visualized. The density of each marker present in each sample assay will be visualized for QC verification. MDS embeddings will be computed for each sample and visualized for sample‐wise distance to observe intra‐ and intergroup distances. For each sample, the median expression of the markers will be computed, and the median expression of all samples will be hierarchically clustered and visualized on a heatmap. Sample‐wise boxplots will also be plotted. If there is more than one group of samples submitted in a project, a group‐wise boxplot will be returned to the user. Non‐redundancy scores will be computed for each marker in each sample to identify the most variable markers across samples [39].

Integrative dimension reduction and clustering. For multiple sample projects, batch‐corrected samples will be merged before dimension reduction. FlowSOM [40] will be used to first carry out the first level of dimension reduction and clustering on the batch‐corrected samples by reducing the cell dimension to 100 dimensions. This forms a set of 100 meta clusters, each containing a set of highly similar cells in terms of the provided markers. Clustering is then carried out on this dimensionally reduced matrix using the consensus clustering (CC) method to estimate a set of clustering results. The Elbow method is used to determine the final cluster number based on the CC output. To visualize every cell in a 2D space, PCA embeddings are calculated, followed by UMAP. The clustering result will be mapped onto both PCA and UMAP embeddings and visualized. For the meta clusters computed using FlowSOM, these SOM clusters will also be dimensionally reduced to UMAP and *t*‐SNE to visualize the goodness of meta‐clustering before final clustering.

#### Phenotypic discoveries

After clustering, the median expression of the markers for each cluster will be computed and visualized in terms of a heatmap and boxplots. Marker densities across cells in each sample will be plotted to visualize marker expression globally. The proportions of samples contributing to each cluster will also be plotted. Cell identities for each cluster and each meta‐cluster will be predicted by computing the phenotypes of each cluster. For each marker, the median expression of each marker in each cluster will be compared against the median expression of the marker in the data. Markers with fold‐change > 1. 25 will be marked as phenotypic markers for the cluster. The list of a final set of markers, for example, CD3+CD4+ for cluster 1, will be the predicted phenotypes for the particular cluster.

### Flow cytometry analysis framework

The analysis is catered for running raw flow cytometry*. fcs* files. Submission of multiple samples is supported (Figure S5).

#### QC and gating strategies

FCS format files will be read in, and scaling will be performed by arcsine transform of the expression matrix of each sample with a co‐factor of 150. The density of each marker present in each sample assay will be visualized for QC verification. Automated gating will be carried out to remove debris or doublets. The gating procedures are as follows, and their purpose is to remove debris or doublets: FSC‐A vs. SSC‐A, FSC vs. FSC‐H, SSC‐W vs. SSC‐H, and FSC‐W and FSC‐H subsequent binary gating approaches. Each gating step will be visualized, and a gating strategy summary table will be provided to the user. After gating, the density of each marker present in each sample assay will be reassessed for QC check. Similar to CyTOF analysis, for each sample, the median expression of the markers will be computed, and the median expression of all samples will be hierarchically clustered and visualized on a heatmap. MDS embeddings will be computed for each sample and visualized for sample‐wise distance, to observe intra and intergroup distances. Non‐redundancy scores are computed for each marker in each sample to identify highly variable markers across the samples.

#### Integrative dimension reduction and clustering

For the multiple samples project, samples will be merged before dimension reduction. Similar to CyTOF analysis, FlowSOM is used to first meta‐cluster the cells to reduce the cell dimension to 100 dimensions. The set of 100 meta clusters will each contain a set of highly similar cells with similar expressions. Clustering is then carried out on this dimensionally reduced matrix using the consensus clustering (CC) method to estimate a set of clustering results. The Elbow method is used to determine the final cluster number based on the CC output. To visualize every cell in a 2D space, PCA embeddings are calculated, followed by UMAP. The clustering result will be mapped onto both PCA and UMAP embeddings and visualized.

#### Phenotypic discoveries

After clustering, the median expression of the markers for each cluster will be computed and visualized in terms of a heatmap and boxplots. Marker densities across cells in each sample will be plotted to visualize marker expression globally. The proportions of samples contributing to each cluster will also be plotted.

### Spatial transcriptomics analysis framework

The analysis is performed for the post‐quantification spatial transcriptomics assay from 10X Genomics. Direct output from Cellranger will be used as input to this analysis framework. An integrative analysis is supported for the submission of multiple samples. A multi‐modal integrative analysis is also supported but, at present, only supports one sample multi‐modal integration (Figure S3).

#### QC metrics assessments and data processing

Using seurat, for each sample, the number of RNA molecule counts for each spot will be calculated and visualized in a violin plot and on the imaging data provided. Normalization is performed using *sctransform* in seurat with a regularized negative binomial model of gene expression to normalize for technical variations. Highly variable genes across the spots for each sample will be computed and visualized on the imaging data.

#### Dimension reduction, clustering, and DE analysis

Since this is essentially single‐cell transcriptomics data, similar dimension reduction and clustering steps are performed compared to the scRNA‐seq workflow. For each sample, PCA will be performed on the sctransformed data, followed by UMAP projections based on the first 30 PCA embeddings. The k‐NN and SNN are computed based on the first 30 PCA embeddings, and clustering is performed via modular optimization using the original Louvain algorithm. Post‐dimension reduction and clustering results will be visualized on UMAP embeddings, and at the same time, clustering information will be mapped onto the spatial imaging data to visualize the location of the clusters on the sample. For each cluster, DE analysis will be performed to look for DE genes in each cluster. For each comparison, genes that could not be detected at a minimum of 0.25 in proportion in either of the two groups of cells are discarded. The final list of DEGs for each cluster is retained by a Bonferroni‐corrected threshold of adjusted *p* < 0.1 and >0.25 on average log2Fold‐Change. DEGs for each cluster will be shown to the user to aid cell‐type annotation. Automated cell type annotation will also be performed using SingleR.

#### Multiple sample analysis

For a project with multiple samples, samples will be merged to perform dimension reduction and clustering, and the same procedure will be carried out as mentioned in the previous step.

#### Multimodal integrative analysis

For a project with both spatial transcriptomics and scRNA‐seq samples provided, similar steps will be carried out compared to the multi‐modal integrative analysis in scImmune Profiling. The scRNA‐seq sample will be read in, and cells with <200 or >6000 genes expressed in one cell will be discarded. This is fixed and catered for 10X Genomics output at the moment. Post‐filtering data are then normalized using the same sctransformed method as the spatial samples. PCA is run followed by UMAP, which uses the first 30 PCA embeddings. Clustering will be carried out based on the first 30 PCA embeddings using k‐NN followed by SNN and modular optimization using the original Louvain algorithm. Automated cell annotation will be carried out to predict the cell‐type identity of each cluster, and seurat integration will be performed by first identifying a set of transfer anchor cells between the spatial data and the scRNA‐seq data, followed by a transfer of the predicted cell type labels in the scRNA‐seq data onto the spatial sample. For each scRNA‐seq‐predicted cell type, the prediction scores for each spatial cluster will be visualized.

## AUTHOR CONTRIBUTIONS

**Lu Pan**: Conceptualization; investigation; funding acquisition; writing—original draft; methodology; visualization; writing—review and editing; formal analysis; software; project administration; data curation; supervision; resources. **Bufu Tang**: Methodology; writing—review and editing. **Xuan Zhang**: Methodology; writing—review and editing; project administration. **Paolo Parini**: Writing—review and editing; project administration; resources. **Roman Tremmel**: Methodology; software; data curation; visualization; writing—review and editing. **Joseph Loscalzo**: Conceptualization; methodology; supervision; validation; writing—review and editing; Writing—original draft; Investigation. **Ákos Végvári and Volker M. Lauschke**: Conceptualization; investigation; writing—original draft; writing—review and editing; validation. **Bradley A. Maron**: Writing—review and editing. **Paola Paci**: Writing—review and editing. **Ingemar Ernberg**: Writing—review and editing. **Nguan Soon Tan**: Writing—review and editing. **Ákos Végvári**: Writing—review and editing. **Zehuan Liao**: Writing—review and editing. **Sundararaman Rengarajan**: Writing—review and editing. **Roman Zubarev**: Writing—review and editing. **Yuxuan Fan**: Writing—review and editing; Methodology. **Xu Zheng**: Writing—review and editing. **Xinyue Jian**: Writing—review and editing. **Ren Sheng**: Writing—review and editing; conceptualization. **Zhenning Wang**: Project administration. **Xuexin Li**: Writing—review and editing; writing—original draft; Conceptualization; investigation; funding acquisition; methodology; project administration; supervision; resources.

## CONFLICT OF INTEREST STATEMENT

Volker M. Lauschke is CEO and shareholder of HepaPredict AB, cofounder, and shareholder of PersoMedix AB, and discloses consultancy work for Enginzyme AB. The other authors declare that they have no competing interests.

## ETHICS STATEMENT

No animals or humans were involved in this study.

## Supporting information


**Figure S1.** Trends of single‐cell omics and their tools in the past decades.
**Figure S2.** List of additional analytic tools provided by the web server.
**Figure S3.** Analysis workflow for the spatial transcriptomics framework.
**Figure S4.** Analysis workflow for the CyTOF framework.
**Figure S5.** Analysis workflow for flow cytometry framework.

**Table S1.** Comprehensive comparison of single cell analyst over other single‐cell webservers.
**Table S2.** Runtime performance benchmarking and docker configuration guidelines for single‐cell multi‐omics workflows.
**Table S3.** Computational resource recommendations for single‐cell multi‐omics analysis by data scale.
**Table S4.** Core R packages and versions for single‐cell multi‐omics bioinformatics workflows.
**Table S5.** Reference R packages (with versions and URLs indicated) used in the analyses.
**Table S6.** Core tools and databases used by the single cell analyst platform.

## Data Availability

The data that supports the findings of this study are available in the supplementary material of this article. Online version of the Single Cell Analyst is available at: www.singlecellanalyst.org. Dockerized version of the webserver and the corresponding code is maintained at the GitHub repository: https://github.com/singlecellanalyst/SCAWebserver. The data and scripts used are saved in GitHub: https://github.com/singlecellanalyst/SCAWebserver. A list of packages used by the platform is available in Supplementary Table 3. Datasets used for demonstration in the workflows on the SingleCellAnalyst platform can be found in the 10X Genomics data set website: www.10xgenomics.com/datasets. Supplementary materials (figures, tables, graphical abstract, slides, videos, Chinese translated version, and update materials) may be found in the online DOI or iMeta Science http://www.imeta.science/.
