# Supplementary material for: Comprehensive analysis of multi‐omics single‐cell data using the single‐cell analyst
Source: Imeta. 2025 Apr 28;4(3):e70038. doi: 10.1002/imt2.70038 (PMC12130572; doi:10.1002/imt2.70038)
Supplement: Supplementary file 1 — Figure S1. Trends of single‐cell omics and their tools in the past decades. Figure S2. List of additional analytic tools provided by the web server. Figure S3. Analysis workflow for the spatial transcriptomics framework. Figure S4. Analysis workflow for the CyTOF framework. Figure S5. Analysis workflow for flow cytometry framework. [file IMT2-4-e70038-s002.docx]

**Comprehensive analysis of multi-omics single-cell data using the single cell analyst**

**Running title**: Comprehensive multi-omics analysis with single cell analyst

Lu Pan^1#^, Bufu Tang^2#^, Xuan Zhang^3#^, Paolo Parini^4^, Roman Tremmel^5^, Joseph Loscalzo^6^, Volker M. Lauschke^7^, Bradley A. Maron^6^, Paola Paci^8^, Ingemar Ernberg^9^, Nguan Soon Tan^10,11^, Ákos Végvári^12^, Zehuan Liao^9,10^, Sundararaman Rengarajan^13^, Roman Zubarev^12^, Yuxuan Fan^14^, Ren Sheng^15,16*^, Zhenning Wang^17,18,19*^, Xuexin Li^20*^

^1^Institute of Environmental Medicine, Karolinska Institutet, Solna 17165, Sweden

^2^Department of Radiation Oncology, Zhongshan Hospital Affiliated to Fudan University, Shanghai 200032, China

^3^Department of Colorectal surgery, Yunnan Cancer Hospital, The Third Affiliated Hospital of Kunming Medical University, Yunnan 650000, China

^4^Cardio Metabolic Unit, Department of Medicine, and Department of Laboratory Medicine, Karolinska Institutet, Stockholm 141 86, Sweden

^5^University of Tuebingen, Tuebingen 72076, Germany

^6^Department of Medicine, Brigham and Women's Hospital, Harvard Medical School, Boston, MA 02115, USA

^7^Department of Physiology and Pharmacology, Karolinska Institutet, Solna 17165, Sweden

^8^Department of Computer, Control and Management Engineering, Sapienza University of Rome, Rome 00185, Italy

^9^Department of Microbiology, Tumor and Cell Biology, Karolinska Institutet, Solna 17165, Sweden

^10^School of Biological Sciences, Nanyang Technological University, Singapore 637551, Singapore

^11^Lee Kong Chian School of Medicine, Nanyang Technological University Singapore, Singapore 308232, Singapore

^12^Department of Medical Biochemistry and Biophysics, Karolinska Institutet, Solna, 17165, Sweden

^13^Department of Physical Therapy, Movement & Rehabilitation Sciences, Northeastern University, Boston, MA 02115, USA

^14^China Medical University-The Queen’s University of Belfast Joint College, China Medical University, Liaoning 110122, China

^15^College of Life and Health Sciences, Northeastern University, Liaoning 110819, China

^16^School of Basic Medical Sciences, Guangzhou Medical University, Guangdong 510000, China

^17^Key Laboratory of Precision Diagnosis and Treatment of Gastrointestinal Tumors, Ministry of Education, China Medical University, Liaoning 110122, China

^18^Institute of Health Sciences, China Medical University, Liaoning 110122, China

^19^The First Affiliated Hospital of China Medical University, Liaoning 110001, China

^20^Department of General Surgery, The Fourth Affiliated Hospital, China Medical University, Liaoning 110032, China

^#^These authors contributed equally: Lu Pan, Bufu Tang, Xuan Zhang

*Corresponding author: [shengren@mail.neu.edu.cn (Ren](mailto:shengren@mail.neu.edu.cn(Ren) Sheng), [znwang@cmu.edu.cn (Zhenning](mailto:znwang@cmu.edu.cn(Zhenning) Wang), [xuexin.li@ki.se](mailto:xuexin.li@ki.se) (Xuexin Li)


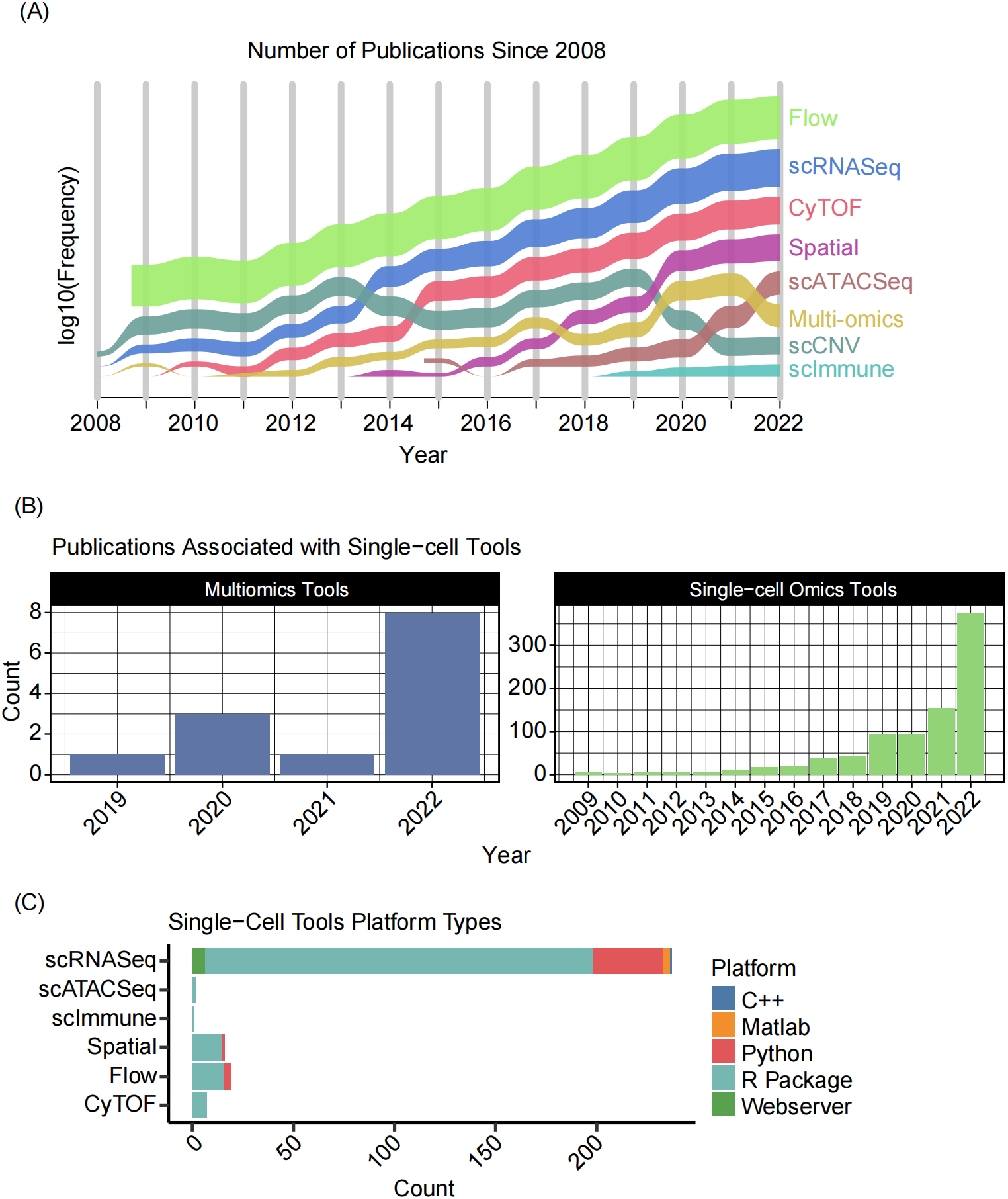


**Figure S1 Trends of single-cell omics and their tools the past decades.** (A) Number of publications (in log_10_ scale) from PubMed associated with various single-cell omics types since 2009. (B) Number of published single-cell tools and single-cell multi-omics tools since 2009. (C) Availability of these tools across different platforms.


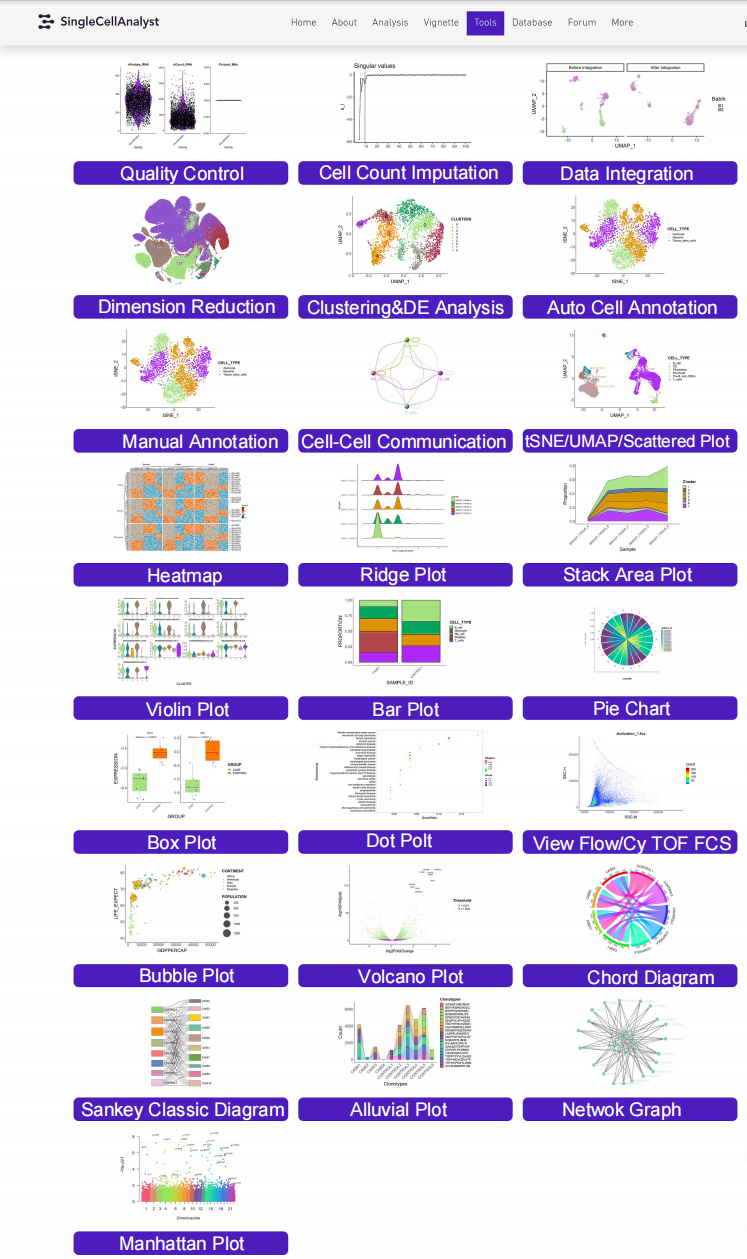


**Figure S2 List of additional analytic tools provided by the web server.** All tools are user-adjustable followed by vibrant visualization outputs.


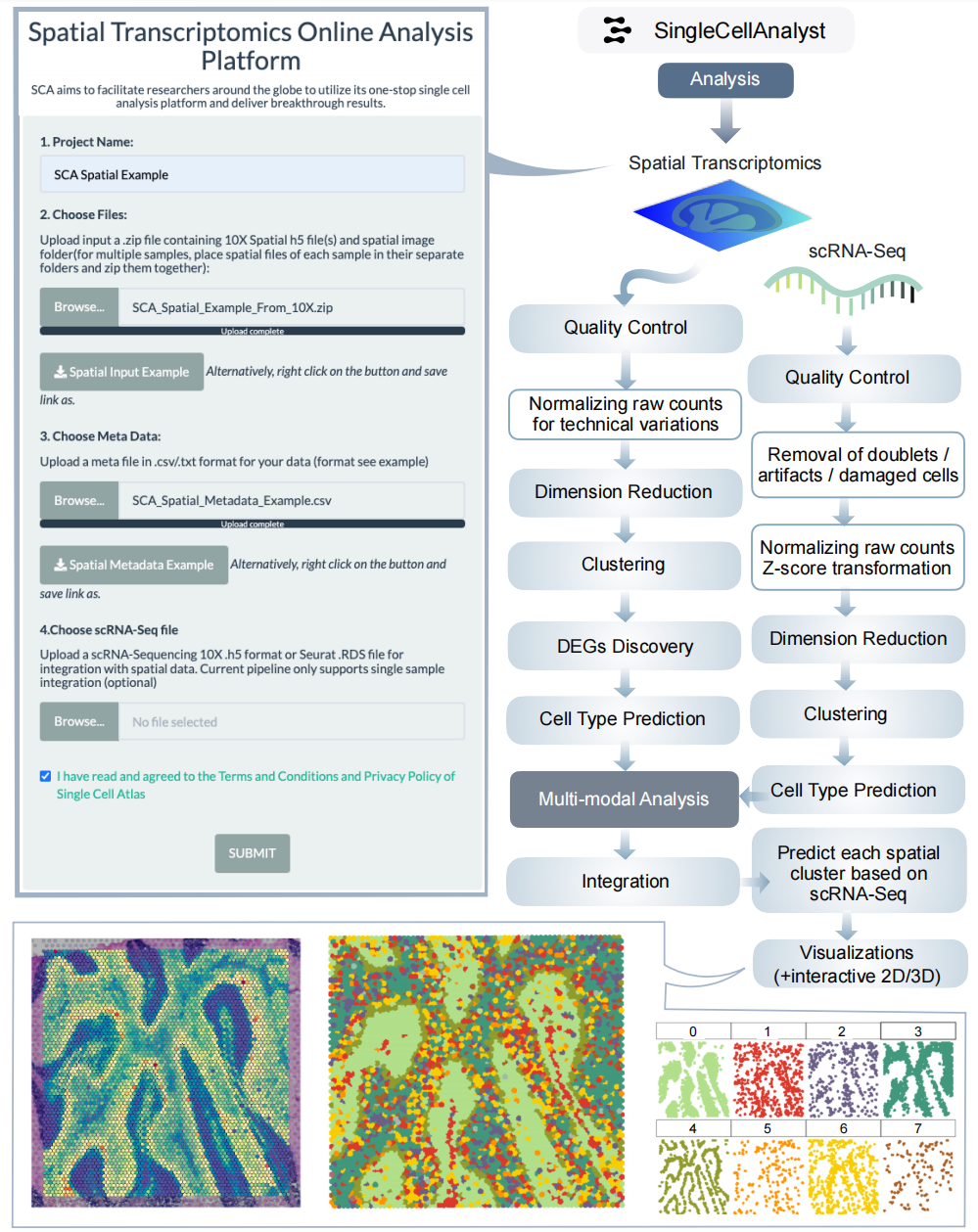


**Figure S3 Analysis workflow for the spatial transcriptomics framework.** Key steps in the workflow for spatial transcriptomics analysis on the multi-omics server.


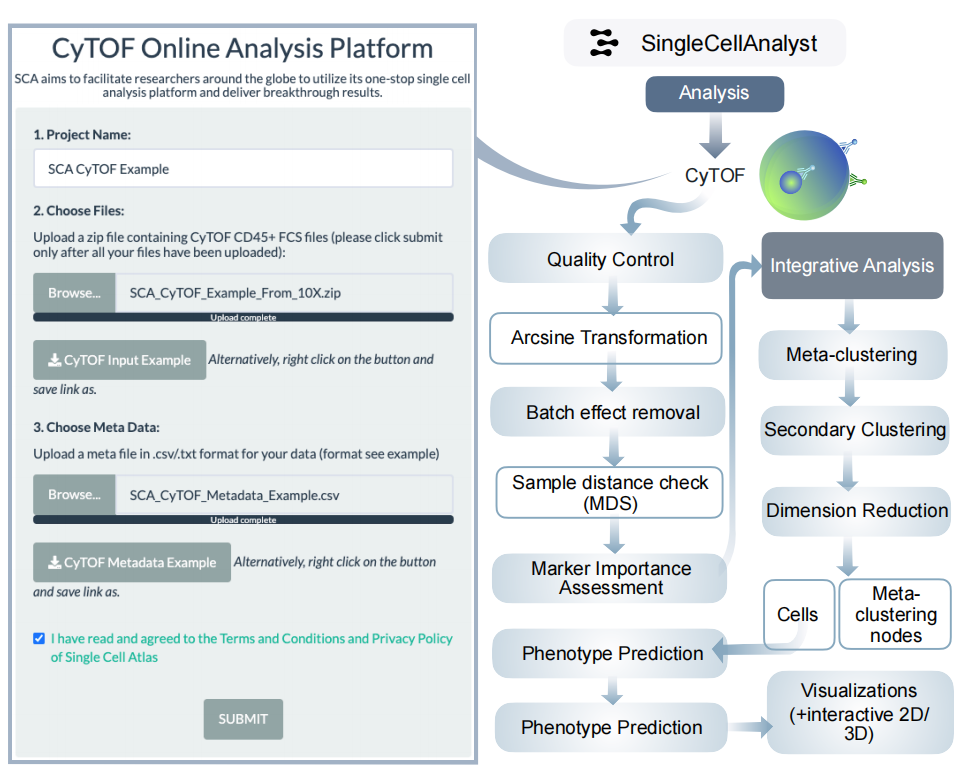


**Figure S4 Analysis workflow for the CyTOF framework.** Key steps in the workflow for CyTOF analysis on the multi-omics server.


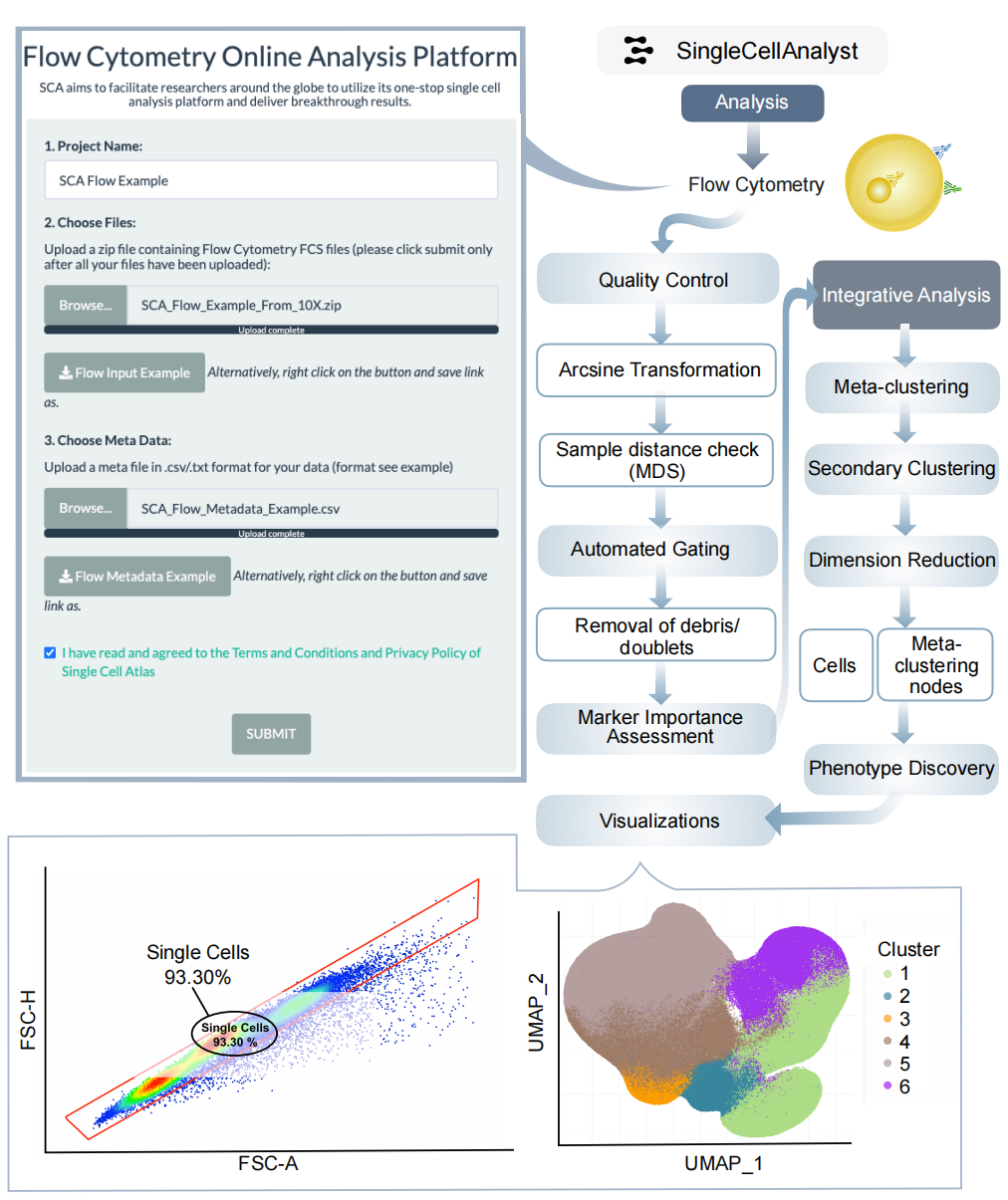


**Figure S5 Analysis workflow for flow cytometry framework.** Key steps in the workflow for flow cytometry analysis on the multi-omics server.
